# *Dioscorea alata* Attenuates Renal Interstitial Cellular Fibrosis by Regulating Smad- and Epithelial-Mesenchymal Transition Signaling Pathways

**DOI:** 10.1371/journal.pone.0047482

**Published:** 2012-11-08

**Authors:** Shu-Fen Liu, Shan-Yu Chang, Tao-Chen Lee, Lea-Yea Chuang, Jinn-Yuh Guh, Chien-Ya Hung, Tsung-Jen Hung, Yu-Ju Hung, Po-Yi Chen, Pei-fang Hsieh, Yu-Lin Yang

**Affiliations:** 1 Department of Internal Medicine, Kaohsiung Medical University, Kaohsiung, Taiwan; 2 Department of Graduate Institute of Biomedical Science, Chung Hwa University of Medical Technology, Tainan, Taiwan; 3 Kaohsiung Chang Gung Memorial Hospital, Chang Gung University College of Medicine, Kaohsiung, Taiwan; 4 Department of Biochemistry, Kaohsiung Medical University, Kaohsiung, Taiwan; 5 Department of Food Nutrition Chung Hwa University of Medical Technology, Tainan, Taiwan; 6 Department of Public Health, National Taiwan University, Taipei, Taiwan; 7 Department of Optometry, Chung Hwa University of Medical Technology, Tainan, Taiwan; 8 Department of Medical Laboratory Science and Biotechnology, Chung Hwa University of Medical Technology, Tainan, Taiwan; University of Edinburgh, United Kingdom

## Abstract

Renal interstitial fibrosis is characterized by increased extracellular matrix (ECM) synthesis. Epithelial-mesenchymal transition (EMT) in kidneys is driven by regulated expression of fibrogenic cytokines such as transforming growth factor-beta (TGF-β). Yam, or *Dioscorea alata* (DA) is an important herb in Chinese medicine widely used for the treatment of clinical diabetes mellitus. However, the fibrosis regulatory effect of DA is unclear. Thus, we examined TGF-β signaling mechanisms against EMT in rat fibroblast cells (NRK-49F). The characterization of DA water-extracts used various methods; after inducing cellular fibrosis in NRK-49F cells by treatment with β-hydroxybutyrate (β-HB) (10 mM), we used Western blotting to examine the protein expression in the TGF-β-related signal protein type I and type II TGF-β receptors, Smads2 and Smad3 (Smad2/3), pSmad2 and Smad3 (pSmad2/3), Smads4, Smads7, and EMT markers. These markers included E-cadherin, alpha-smooth muscle actin (α-SMA), and matrix metalloproteinase-2 (MMP-2). Bioactive TGF-β and fibronectin levels in the culture media were determined using ELISA. Expressions of fibronectin and Snail transcription factor, an EMT-regulatory transcription factor, were assessed by immunofluorescence staining. DA extract dose-dependently (**50**–200 µg/mL) suppressed β-HB-induced expression of fibronectin in NRK-49F cells concomitantly with the inhibition of Smad2/3, pSmad2/3, and Smad4. By contrast, Smad7 expression was significantly increased. DA extract caused a decrease in α-SMA (α-smooth muscle actin) and MMP-2 levels, and an increase in E-cadherin expression. We propose that DA extract might act as a novel fibrosis antagonist, which acts partly by down regulating the TGF-β/smad signaling pathway and modulating EMT expression.

## Introduction

The incidence of chronic kidney disease (CKD) is rapidly increasing in industrialized countries, partly due to increases in disorders such as obesity, diabetes, and peripheral artery disease [10], [21]. In recent years, researchers have uncovered evidence supporting the medical effects of Chinese-Herbal Medicine; Yam tuber, or *Dioscorea* spp., is considered a herbal medicine in Taiwan.

Tubulointerstitial fibrosis is the common pathway in progressive renal disease; it leads to functional deterioration and eventual loss of renal function irrespective of the diverse initial causes [12], [24], [25]. Tubulointerstitial fibrosis is involved in the accumulation of extracellular matrix components and loss of tubular architecture. Proximal tubular epithelial cells play a central role in renal tubulointerstitial fibrosis [1], [3]. A critical step in the pathogenesis of tubulointerstitial fibrosis is epithelial mesenchymal transition (EMT), whereby renal tubular epithelial cells change phenotypically and functionally into myofibroblasts [26]. The factor most capable of inducing EMT is transforming growth factor-β1 (TGF-β1). The transformation is characterized by the loss E-cadherin expression and increased expression of α-smooth muscle actin (α-SMA). Therefore, the occurrence of EMT in the kidneys provides a significant therapeutic target; it is important to prevent tubular epithelial cells from undergoing EMT to prevent tubulointerstitial fibrosis.

The pathogenesis of kidney fibrosis is characterized by overproduction and deposition of extracellular matrix (ECM) [16], [20]. Extensive studies show that the myofibroblastic activation of glomerular mesangial cells and interstitial fibroblasts, as manifested by α-smooth muscle actin (α-SMA) induction, plays a crucial role in ECM overproduction [2], [18]. TGF-β signaling is transmitted from the cell surface to the nucleus through transmembrane type I and type II serine and threonine kinase receptors, and their downstream mediators (known as Smads). On TGF-β stimulation, Smad2, and Smad3 undergo phosphorylation, triggering an interaction with Smad4 [9], [16], [22]. The Smad complex translocates into the nucleus, where it binds to a specific *cis*-acting element in the regulatory region of the TGF-β target genes (e.g., fibronectin) and directs cell transactivation.

Natural products of plant origin are still a major part of traditional medicinal systems in many countries, however, herbal medicine is often categorized as a form of complimentary or alternative treatment because the lack of scientific and supportive evidence excludes it from mainstream therapy [Cassileth, 1999].Dioscorea alata (DA) in modulation of production of the expression of tumour necrosis factor (TNF)- α, interleukin-1 (IL-1), IL-6 and other inflammatory cytokines in macrophage. (Choi and Hwang, 2002; Liu et al., 2007). In addition, Dioscorea spp. was widely touted to be beneficial for menstrual complaints, perimenopausal symptoms, rheumatoid arthritis and other complaints. (Braun and Cohen, 2007; Carroll, 2006; Soffa, 1996). Previous studies (Chang et al., 2004) demonstrated that D. alata feeding had antioxidant effects and D. atatas exhibited scavenging activities against DPPH and hydroxyl radicals (Hou et al., 2001). Hou et al., (2002) reported that testing anti-DPPH and anti-hydroxyl radicals, reducing powers, and anti-lipid per oxidation activities. However, the Dioscorea alata was responsible for β-HB-mediated fibrosis remains unclear.

We investigated the mechanism underlying reduction in renal fibrosis produced by the Chinese herb *Dioscorea alata* (DA). We show that crude DA aqueous extract contains compounds that provide therapeutic effects for renal fibrosis. These effects involve the antagonization of TGF-β-induced fibrogenic signals (Smad pathways) and EMT processes in interstitial fibroblast cells. Our findings are important for the development of a novel agent against TGF-β signaling and renal interstitial fibrosis.

## Materials and Methods

### Extraction and Isolation of *Dioscorea alata*

*Dioscorea alata* was purchased from the Kaiser Pharmaceutical Company (Tainan, Taiwan). One hundred grams of the dried flower was immersed in distilled water (1000 ml) and boiled for 20 minutes. The solution was then concentrated to 100 ml at 40°C. Particulates were collected by filtration using 325-mesh sieve (Kuang Yang) and lyophilized (Kingmech, FD-4.5-12P).

### Cell Culture

NRK-49F cells (CRL-1570) were obtained from the American Type Culture Collection (ATCC), a normal Rattus norvegicus kidney cell line, was cultured in Dulbecco's modified Eagle's medium (Gibco, Carlsbad, CA) supplemented with 5% bovine calf serum (BCS), 100 U/ml penicillin, and 100 µg/ml streptomycin (HycloneLabs, Logan, UT) at 37°C under 5% CO_2_. The cells were trypsinized using 0.05% trypsin-EDTA (Hyclone).

### Scattering Assay

The protocol was performed according to Chang HY et al, 2011. Cells (1×10^5^) were seeded in each well of a 6-well plate and incubated overnight in a 37°C incubator with 5% CO2. Cells were treated with culture medium including on contraction of β-HB (10 mM) and/or DA. Cells were taken at 200× magnification. Four independent experiments were conducted, and the data were analyzed with t test using GraphPad Prism 5. The distance between groups was considered to be statistically significant when p<0.05.

### Western Blot Analysis

Western blot assay was used to evaluate protein expression of the RI and RII TGF-β receptors, and their downstream signal transducers (e.g., Smad4 and Smad7). In brief, cells were lysed using lysis buffer (10 mM Tris, 1 mM EDTA, 1% Triton X-100, 1 mM Na_3_VO_4_, 20 µg/ml aprotinin, 20 µg/ml leupeptin, 1 mM dithiothreitol, and 50 µM PMSF). The crude protein lysate was resolved by 10% sodium dodecyl sulphate-polyacrylamide gel electrophoresis (SDS–PAGE) under reducing conditions and transferred to polyvinylidene difluoride membranes (Millipore, Bedford, MA). Samples were then blocked with 10% (w/v) non-fat milk in Tris buffer saline tween (TBS-T) for 1 h at 37°C. Individual membranes were probed with a 1 ∶ 2000 (v/v) ratio of rabbit polyclonal antibodies to anti-Smad2/3 (sc-8332), anti-pSmad2/3 (sc-11769), anti-Smad4 (sc-7154), anti-Smad7 (sc-11392), anti-E-cadherin (sc-7870), anti-MMP-2 (sc-10736), anti-TβRI (sc-9048), anti-TβRII (sc-1700; Santa Cruz Biotechnology, Santa Cruz, CA), or Snail (3879; Cell Signaling Technologies, Beverly, CA); and a 1 ∶ 2000 ratio of anti-ß-actin (Sigma-Aldrich, St Louis, MO A-5316), or mouse monoclonal antibodies, to α-SMA (sc-32251; Santa Cruz). After hybridization at 37°C, blots were washed and hybridized with 1∶4000 (v/v) dilutions of goat anti-rabbit IgG or horseradish peroxidase-conjugated secondary antibody (Jackson ImmunoResearch Laboratories, West Grove, PA) or donkey anti-mouse IgG- or horseradish peroxidase (Santa Cruz). The signals were visualized using enhanced chemiluminescence (ECL) and X-ray film (Konica, Tokyo, Japan), with β-actin as an internal control.

### Enzyme-Linked Immunosorbent Assay (ELISA)

ELISA was used to evaluate the expression of secreted fibronectin and TGF-β1. To quantify fibronectin or TGF-β1 in the supernatant of cultured NRK-49F cells, conditioned culture medium was collected and centrifuged at 1200 rpm for 5 min to remove particulates. The clear supernatant was then collected and concentrated, then stored at −80°C for further use. A commercial sandwich enzyme-linked immunosorbent assay kit was used for detection of extracellular fibronectin (Takara Bio, Inc., Shiga, Japan) or TGF-β1 (R&D Systems, Minneapolis, MN). Detection was performed according to the manufacturer's instructions. Sample absorbances at 450 nm were analyzed using an ELISA reader, and the concentration of each sample determined by interpolation with a standard curve, generated using an exogenous fibronectin (12.5, 25, 50, 100, 200, 400, 800 ng/ml) or TGF-β1 (0–2,000 pg/ml) as the standard.

### Immunofluorescene Staining

Cells were cultured in glass chamber slides (Nunc, Rochester, NY) then fixed with 4% paraformaldehyde for 10 min, permeabilized with 0.1% Triton X-100 in phosphate-buffered saline (PBS). After incubation blocked with 1% bovine serum albumin in PBS for 1 hr. The Cells were incubated with primary antibody overnight, washed with PBS, and incubated with the FITC-conjugated secondary antibody for 1 hr. The slides were mounted with Fluorescent Mounting Medium (Santa Cruz), and observed with a fluorescence microscope (Olympus, Japan).

### Cell Viability Assay

Cell viability in NRK-49F cell line was assessed by Trypan blue exclusion assay. Trypan blue was added to the medium and the percentage of viable cells was determined by counting on a hemocytometer the number of cells able to exclude the dye. Percentage viability was calculated using the formula:
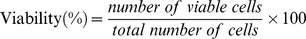



### Cell proliferation by MTT assay

MTT assays were performed to evaluate the cell viability of NRK-49F cells. Cells (1×10^4^ cells/dl) were plated and incubated for 24 hrs in wells of a 96-well plate. Then treated with culture medium including on contraction of β-HB (10 mM) and/or DA. After 24-hrs incubation, 10 µl of sterile MTT dye were added, and the cells were incubated for 6 hrs at 37°C. Then, 100 µl of acidic isopropanol (0.04 M HCl in isopropanol) were added and thoroughly mixed. Spectrometric absorbance at 595 nm (for formazan dye) was measured with the absorbance at 655 nm for reference.

### LDH Assay for Cytotoxicity

Cells were maintained and passaged as described above. The cells were seeded in 96 well plates at a density of 2×10^4^ cells/well in complete medium and incubated at 37°C in 5% CO^2^ overnight. Supernatant from the conditioned cells was collected and stored. Supernatant from maintained cells treated with 1% Triton X-100 was regarded as a positive control for maximum lactate dehydrogenase (LDH) release. After 24 h incubation at 378C in 5% CO^2^, the supernatants were collected and centrifuged at 4,500 g for 5 min to remove contaminating cells, and the level of LDH measured in duplicate using a cytotoxicity detection kit (Clontech, CA, US)

### Statistical Analysis

All data are presented as means ± SD. The unpaired Student's *t*-test was used for comparison of two groups. A *P* value<.05 was considered to be statistically significant.

## Results

**Figure 1:**
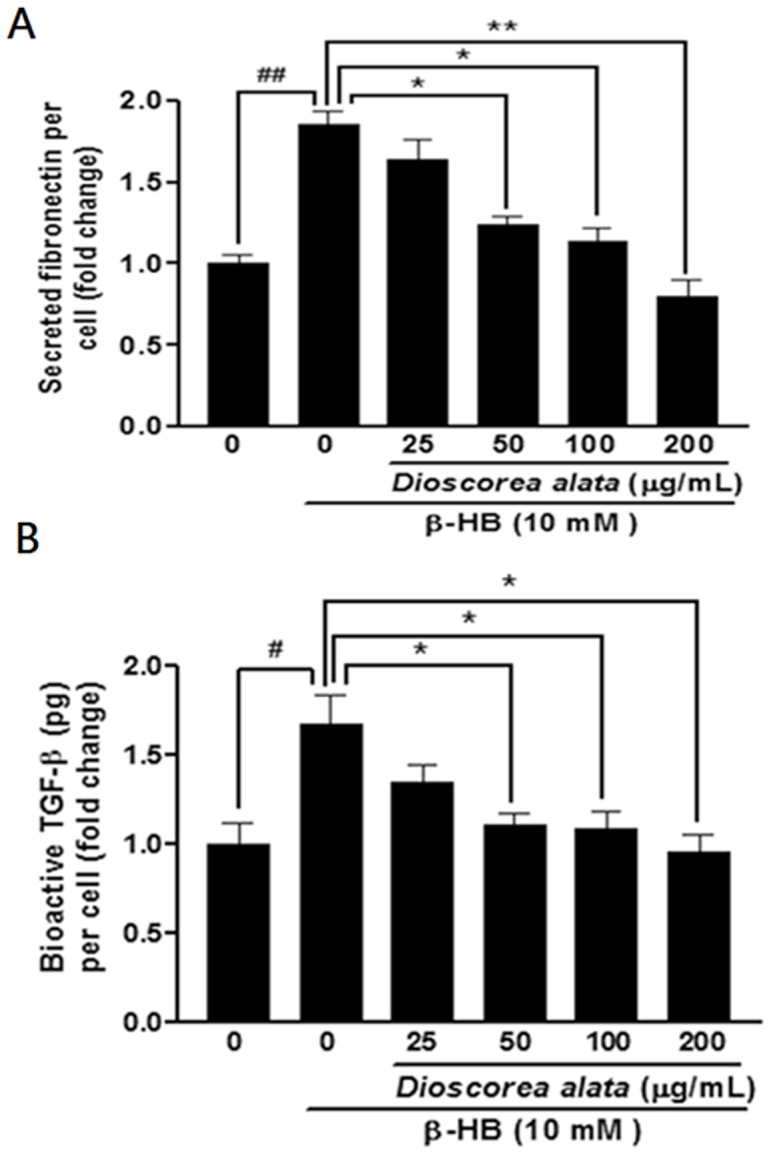
Effects of DA extract on levels of secreted fibronectin and bioactive TGF-β. A, B: Cells were treated with β-HB (10 mM) in 5% FCS for 48 h, followed by treatment with DA water extract for a further 24 h. The supernatant was collected and subjected to fibronectin and TGF-β1 ELISA analysis. The secreted level for each experimental condition was normalized according to cell number. Measurements were repeated twice and similar results obtained. The figure shows that β-HB induced significant increases in fibronectin and TGF-β levels. Additionally, the DA extract dose-dependently reduced β-HB-induced increase in fibronectin and TGF-β secretion in NRK cells. ^##^ *P*<0.01, *^#^P*<0.05, **P<0.01, *P<0.05.

**Figure 2:**
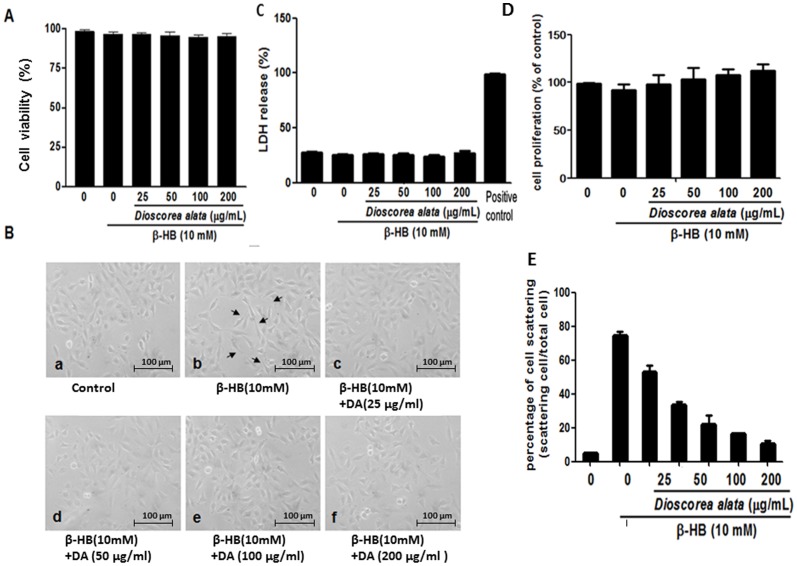
Effects of DA extract on the cellular viability and cell morphological transformations. NRK-49F cells were treated with β-HB (10 mM) in 5% FCS for 48 h, followed by treatment with DA water extract (0, 25, 50, 100 and 200 µg/ml) for a further 24 h. A: The remaining cell layer was subjected to trypan blue exclusion assay. The figure shows that DA extract does not affect the viability of cultured cells. B: Cells were manipulated as above. (a) Control group (b) β-HB (10 mM) treating group. (c) β-HB (10 mM) treating group with 25 µg/ml DA. (d) β-HB (10 mM) treating group with 50 µg/ml DA. (e) β-HB (10 mM) treating group with 100 µg/ml DA. and (f) β-HB (10 mM) treating group with 200 µg/ml DA. (C) The supernatant was collected and subjected to LDH assay. (D) Cell proliferation was determined by MTT assay. (E) Effects of DA extract on the cell morphological transformations was determined by scattering assay (n = 4, p<0.05).

We further investigated the fibrosis-regulatory effects of DA extract on renal fibroblast cells. β-hydroxybutyrate (HB)-induced production of fibronectin and TGF-β provided an *in vitro* model for renal fibrosis. As shown in Fig. 1, β-HB (10 mM) significantly increases extracellular fibronectin (*P*<0.05) and bioactive TGF-β (*P*<0.05) concentrations. Diabetes is usually concomitant with ketonuria and end-stage renal fibrosis, thus we used β-HB (the major form of ketones in humans) to induce renal cellular fibrosis. DA water extract (25, 50, 100, and 200 µg/ml) dramatically suppressed β-HB-induced increases in both fibronectin and bioactive TGF-β in a dose dependent manner. Because TGF-β is a potent fibrogenic cytokine inducer for renal fibroblasts, it is reasonable to assume that DA extract may contain certain active compounds with potential for fibrosis-inhibition. To clarify the influence of DA extract on cellular viability, MTT and LDH were performed (Fig. 2C and 2D). We found that DA water extract does not affect the viability of cultured fibroblasts. Thus, DA extract does not exert its effects by inhibiting cell growth or by inducing cell death. Moreover, cellular scattering was significantly and dose-dependently (i.e. 50, 100, 200 µg/mL) restored compared with β-HB treating group (n = 4, p<0.05).

**Figure 3:**
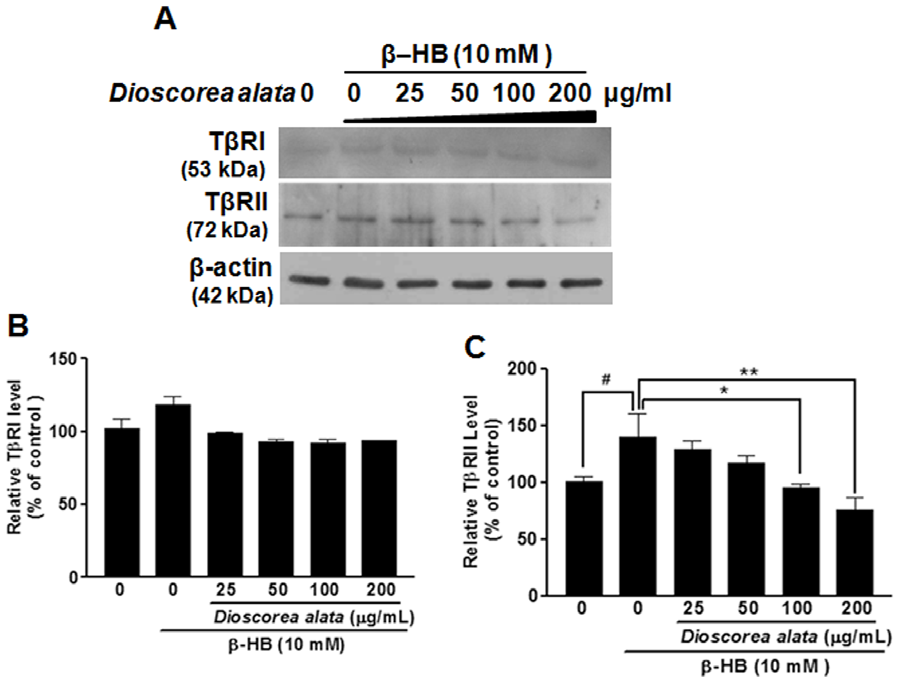
Effects of DA extract on β-HB-induced TGF-β receptor expression in NRK cells. (**A**): NRK Cells were treated with β-HB (10 mM) in 5% FCS for 48 h, followed by treatment with DA water extract of for another 24 h. For Western blot analysis (A), cell extracts were subjected to SDS-PAGE and immunoblotted with primary antibodies against TβRI and TβRII. β-actin was used as a internal control. The expression of TβRI (**B**) and TβRII (**C**) proteins was normalized to that of β-actin. Results are expressed as means ±SD of three observations. DA extract significantly reduced the β-HB-induced increases in the TβRII expression level. ^#^ *P*<0.05, *P<0.05, **P<0.01.

To elucidate the underlying mechanism by which DA regulates renal cellular fibrosis, the expressions of two TGF-β receptor types (TβRI and TβRII) were investigated. The presence of these receptors strongly correlates with susceptibility to cellular fibrosis. As shown in Fig. 3, β-HB induced a significant increase in the level of type II TGF-β receptors. Most importantly, Western blot analysis showed a statistically significant reduction in protein expression of TβRI instead of TβRII for the DA aqueous extract. These results, together with the results shown in Fig. 1D suggest that DA extract has the potential to retard renal cellular fibrosis via down-regulation of the TGF-β autocrine loop, decreasing both TGF-β secretion and the expression of TGF-β receptors.

**Figure 4:**
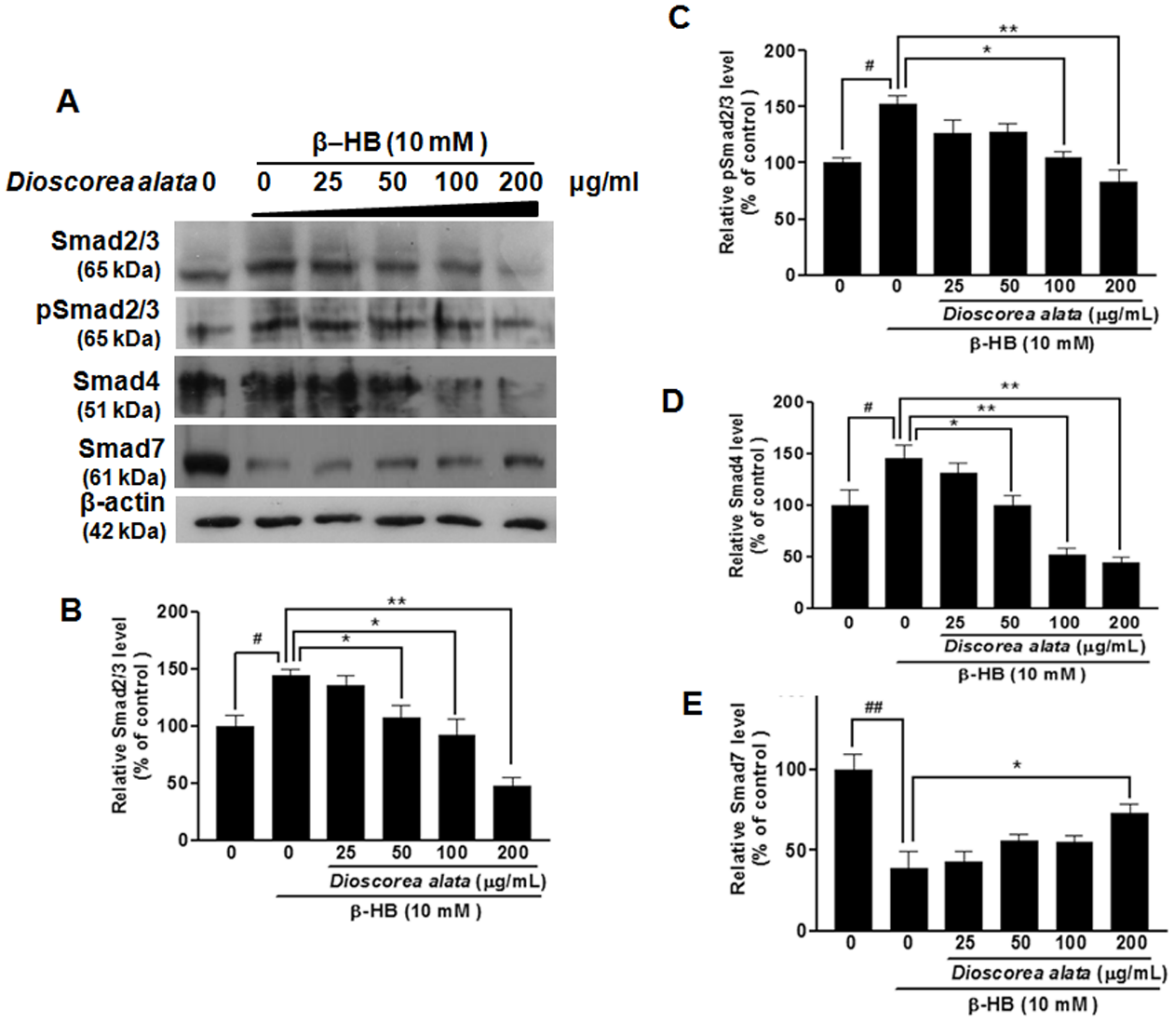
Alteration of cellular levels of Smad2/3, pSmad2/3, Smad4, and Smad7 in NRK cells exposed to β-HB then treated with DA extract. NRK Cells were treated with β-HB (10 mM) in 5% FCS for 48 h, followed by treatment with DA water extract for another 24 h. A: Western blot analysis; cell extracts were subjected to SDS-PAGE and immunoblotted with primary antibodies against Smad2/3, pSmad2/3, Smad4, and Smad7. β-Actin protein was used as an internal control. B–E: Data were scanned and normalized to β-actin. β-HB treatment resulted in increased expression of Smad2/3, pSmad2/3 and Smad4, and a decrease in Smad7 expression; importantly, DA extract significantly reversed β-HB-induced increases in Smad2/3, pSmad2/3 and Smad4, and the concomitant decrease in Smad7. ^##^ *P*<0.01, *^#^P*<0.05, **P<0.01, *P<0.05.

**Figure 5:**
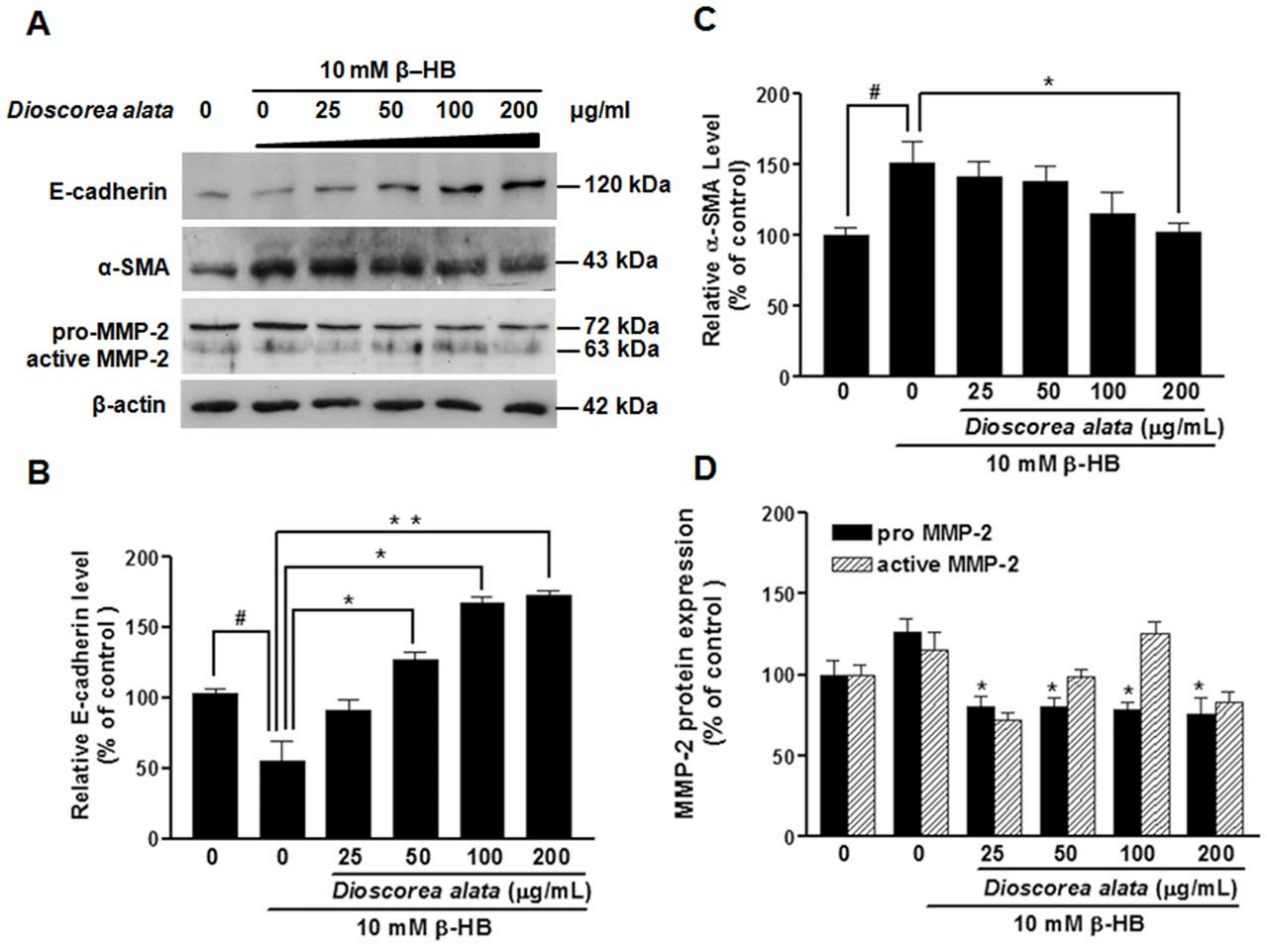
Immunoblot analyses of E-cadherin: α-SMA and MMP-2. NRK-49F Cells were treated with β-HB (10 mM) in 5% FCS for 48 h, followed by treatment with DA water extract for a further 24 h. For Western blot analysis (A), cell extracts were subjected to SDS-PAGE and immunoblotted with primary antibodies against E-cadherin, a-SMA, and MMP-2. β-actin was the internal control. B–D: Densitometry analysis from A is shown. It is evident that β-HB induced an increase in α-SMA and MMP-2, and caused a decrease in E-cadherin expression; DA reversed the β-HB -induced increase in α-SMA and MMP-2, and also reversed the decrease in E-cadherin expression. The experiment was duplicated with similar results observed. ^#^ *P*<0.05, **P<0.01, *P<0.05.

Post-receptor regulation of TGF-β was examined, since the Smad family is the most important mediator for TGF-β signaling. As shown in Fig. 4B–D, β-HB (10 mM) significantly increased pSmad2/3, Smad2/3, and Smad4 levels. Intriguingly, DA water extract (25, 50, 100, and 200 µg/ml) dramatically suppressed β-HB-induced increases in pSmad2/3, Smad2/3, and Smad4 in a dose-dependent manner. These observations show that DA may reverse β-HB-induced cellular fibrosis by regulating and suppressing TGF-β down-stream signals. We examined Smad7, a powerful intracellular TGF-β antagonist. Treatment with 10 mM β-HB induced significant decreases in Smad7. The DA water extract dose-dependently (25, 50, 100, 200 µg/ml) and dramatically reversed β-HB-induced decreases in Smad7 (P<0.05, Fig. 5E). In other words, DA extract might ameliorate renal cellular fibrosis by inducing an increase in inhibitory Smad7. We noted that 200 µg/ml DA extract also dramatically decreased Smad4, Smad2/3, and pSmad2/3. Thus, aqueous DA extract has great potential to induce TGF-β signaling and so regulate renal cellular fibrosis.

The morphologies of cells undergoing β-HB induced tubular epithelial-myofibroblast transition were observed through a light microscope. NRK-49F cells displayed the typical cobblestone morphology of epithelial cells when grown in controlled medium alone (Fig. 2a). Treatment with 10 mM β-HB induced clear morphologic changes by 72 h, with elongated cells dissociating from neighboring cells and losing their cobblestone monolayer pattern (Fig. 2b). Simultaneous treatment of NRK cells with β-HB (10 mM) and DA extract (25, 50, 100, and 200 µg/ml) gradually restored the epithelial character of the NRK-49F cells (Fig. 2c–f).

E-cadherin and α-SMA (α-smooth muscle actin) are both tubular epithelial-myofibroblast transition markers, thus we investigated α-SMA and E-cadherin expression in NRK-49F. We found that β-HB suppressed E-cadherin expression in NRK-49F cells. Western blot analyses revealed significant suppression of E-cadherin protein after 10 mM β-HB treatment for 72 h. Incubation with DA extract restored the expression of E-cadherin protein in NRK-49F cells in a dose-dependent manner (Fig. 5B). These results indicate that DA extract not only suppresses the expression of myofibroblast marker α-SMA (Fig. 5C) in NRK-49F cells, but also restores the expression of epithelial marker E-cadherin. Matrix metalloproteinase MMP-2 is a key ECM-degrading enzyme during cell migration. The enzyme is significantly inhibited by DA extract. However, data from our MMP-2 study demonstrates that β-HB (10 mM) increases pro-MMP-2 levels (Fig. 5D).

**Figure 6:**
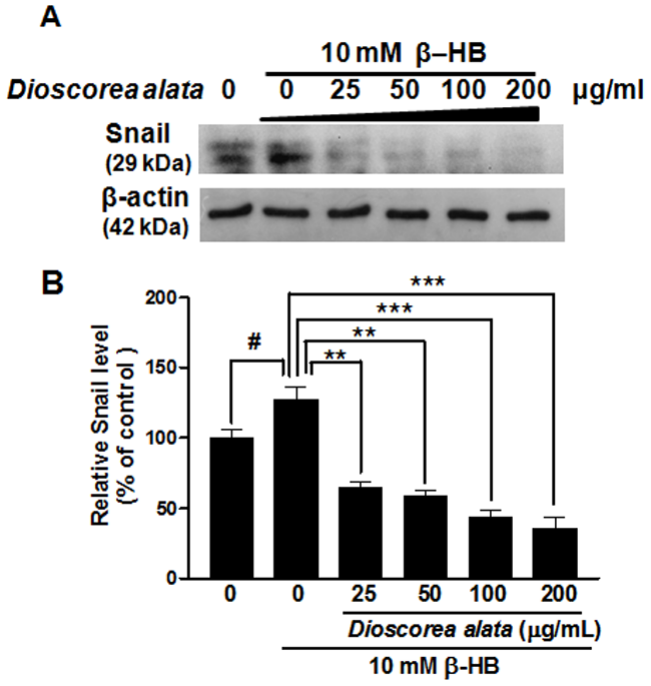
Western blot analysis of Snail transcription factor expression in NRK-49F cells. NRK-49F Cells were treated with β-HB (10 mM) in 5% FCS for 48 h, followed by treatment with DA water extract for another 24 h. A: After following NRK-49F Cell culture protocols, SDS-PAGE was performed with cell extracts or culture media. Western blot analysis used primary antibodies against Snail. β-Actin protein was used as an internal control. B: schematic diagram of Snail relative expression levels, Treatment with DA extract decreased the Snail expression induced by β-HB, in a dose-dependent manner. ^#^ *P*<0.05, *P<0.05, **P<0.01.

**Figure 7:**
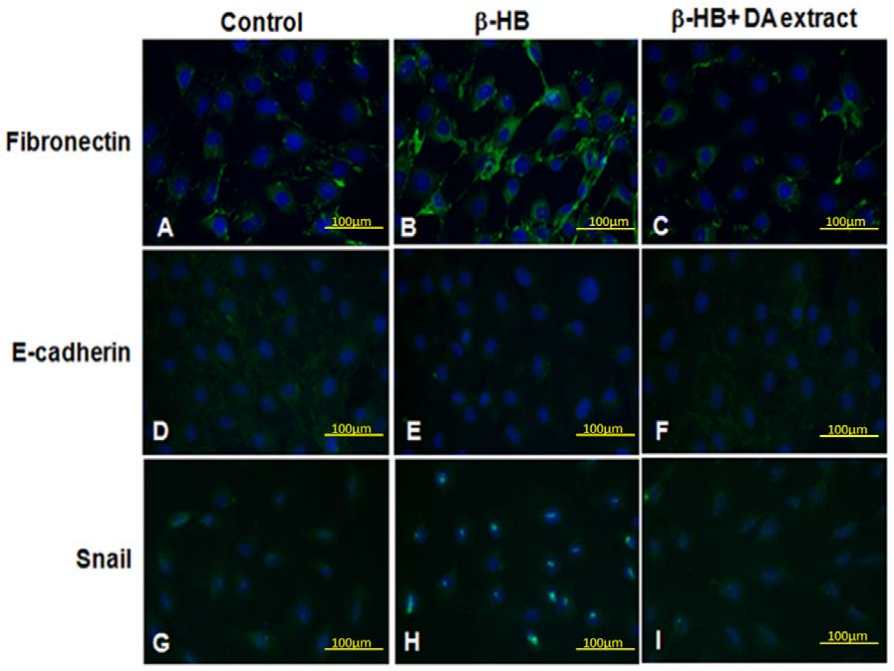
The effects of DA extract NRK-49F cells immunofluorescence stain with antibodies against Fibronectin, E-cadherin, and Snail. Treatment with DA extract decreased the β-HB–induced fibronectin (A–C), Snail (G–I), and the increase E-cadherin (D–F) expression in NRK-49F (a and d) control. B, E, and H: β-HB (10 mM). C, F and I: β-HB and DA extract (200 µg/ml).

Snail is a major transcription factor driving the EMT process. It is upregulated during mesothelial cell EMT in patients treated with peritoneal dialysis [23]. We wondered if this transcription factor is also involved in DA-regulated EMT. Figure 6 shows that exposure of fibroblast to β-HB significantly enhances the expression of Snail protein. However, DA extract at concentrations as low as 25 µg/ml dramatically inhibited Snail. These observations demonstrate that DA extract may regulate renal fibrosis by modulating Snail expression, and this suggestion is supported by Figure 6. Immunofluorescence assay showed that treatment with β-HB resulted in an increase in fibronectin expression (Fig. 7, stained green) and decreased expression of E-cadherin and Snail. Administration of DA extract (200 µg/mL) reduced these changes, almost reversing them. Thus, DA extract might exert renal fibrosis antagonizing effects by regulating the expression of Snail (Fig. 7G–I).

## Discussion

Progression of chronic renal disease, characterized by tubular atrophy and deposition of extracellular matrix proteins in the renal interstitium, is considered an irreversible process leading to end-stage renal failure. Thus, renal interstitial fibroblasts may be an early indicator of nephropathy. In this study, we used β-hydroxybutyrate (β-HB) (<10 mM) to establish a fibrosis model in fibroblasts, because β-HB is the major ketone species present in serum during diabetic ketoacidosis. Blood ketone concentrations can reach 7–8 mM in response to starvation, and can exceed 20 mM during diabetic ketoacidosis. In our study, β-HB treatment (10 mM) induced significant expression of fibronectin in NRK cells (renal interstitial fibroblasts) (Fig. 1C) and bioactive TGF-β1 (Fig. 1D). Treatment with β-HB also resulted in expression of Smad2/3, and Smad4, while Smad7 was dramatically suppressed (Fig. 4). This observation is consistent with our previous findings showing that β-HB-induced collagen production in proximal tubule cells is dependent on TGF-β and Smad3. Thus, this study clarifies the mechanism underlying β-HB-induced renal fibrosis.

Chinese yam (*Dioscorea* spp.) is used by traditional Chinese medicine; the tubers have antioxidant and anti-inflammatory effects [7], [17]. Chang et al. (2004) reported that Chinese yam (D. alata) had potential antioxidant effects. Ken Wojcikowski et al., 2008 showed that Dioscorea villosa exhibited a direct effect to renal cells as well as caused apparent transformation of the surviving renal tubular epithelial cells. Our study demonstrates that DA extract has a protective role against renal cellular fibrosis. Several reports show that yam contains active fractions including mucin, dioscin, diosgenin, allantoin, choline, polyphenol oxidases, and proteinsjavascript∶void(0); that can reduce oxidative and inflammatory stress. The functional significance of renal protection and its mechanism of action against renal fibrosis remain unclear. This study provides convincing evidence that aqueous extracts of DA provide a novel approach to fibrosis control that functions by suppression of TGF-β signaling.

TGF-β is a powerful fibrogenic that promotes the deposition of extracellular matrix and facilitates epithelial-mesenchymal transition [11], [13]. TGF-β Signal transduction is mediated by a conserved mechanism that depends on Smads to transfer the extracellular stimulus into the nucleus. TGF-β converges on the SMAD pathway by recruiting receptor-regulated Smad2 and common-mediated Smad4 [4]. Smad2 inactivation or Smad4 down regulation terminates TGF-β signaling. Thus, developing agents that antagonize TGF-β post-receptor signaling has become a critical issue facing nephropathology research. In the present study, β-HB enhanced the expression of type II TGF-β receptors (Fig. 3) and induced activation of the Smads pathway, increasing Smad2/3, pSmad2/3, and Smad4 while decreasing inhibitory Smad7 (Fig. 4). Further, the DA water extract dramatically attenuated β-HB-induced cellular fibrosis by decreasing the number of type II TGF-β receptors and reducing downstream signaling (including Smad2/3, pSmad2/3, and Smad4). Thus far, there have been few identified inhibitors for type II TGF-β receptors that show clear antifibrotic effects. This study is the first to suggest the potential medical efficacy of DA as an inhibitor for the type II TGF-β receptor for treatment of renal fibrosis. Our results demonstrate that DA extract is a medicinal compound capable of targeting SMAD signaling.

Emerging evidence suggests that EMT is a major event in the pathogenesis of tubulointerstitial fibrosis. In response to the TGF-β1 signal, tubular epithelial cells transdifferentiate to myofibroblasts [4], [6], [19]. The phenotypic conversion involves loss of epithelial polarity, cessation of epithelial E-cadherin expression, disruption of tubular basement membrane, acquisition of spindle-like morphology, *de novo* synthesis of α-SMA, and production of matrix proteins [14], [15]. The appearance of spindle-like morphology, loss of epithelial E-cadherin, and *de novo* α-SMA synthesis are specific EMT markers; at high DA concentrations, EMT inhibition was complete as evidenced by full restoration of epithelial morphology (Fig. 2B).

We found that β-HB increased expression of α-SMA, and decreased E-cadherin expression. DA extract dose-dependently (50, 100, and 200 µg/mL) lessened α-SMA expression, while E-cadherin levels were maintained (Fig. 5). This study is the first to investigate the effects of DA extract on EMT in NRK-49F cells. Our results demonstrated that DA extract produces a remarkable inhibitory effect on β-HB-induced EMT.

These observations propose that DA attenuates renal proximal tubular epithelial cells fibrosis induced by β-HB. TGF-β1 is a key modulator of organ fibrosis following tissue injury [5], and EMT is a critical step in the pathogenesis of tubulointerstitial fibrosis, our findings shed new light on the mechanism by which DA extract elicits renoprotective effects in ameliorating the pathogenesis of tubulointerstitial fibrosis.

DA extract can down-regulate the TGF-β/Smads signaling pathway via suppressing TGF-β1 expression, inhibiting TβR-II expression and function, down-regulating Smad 2/3 phosphorylation, and preventing the down- regulation of Smad7 by TGF-β1. MMP-2 has a role in renal fibrosis, particularly during the early pre-fibrotic stage. However, MMP-2 and MMP-9 are not only stimulated by oxidative stress, more importantly, they are up-regulated by TGF-β1 through signal mediators such as Smads and integrin-linked kinase (ILK) [8]. We also showed that MMP-2 activity increased in NRK-49F cells after incubation with β-HB, but this increase was reversed by treatment with DA extract. Therefore, the TGF-β1/Smads transduction pathway plays a key role in tubular EMT and in DA pharmacological mechanisms against EMT and renal fibrosis.

In vitro experiments used in this study have significantly and clearly explained the rationale. Despite, the results of in vitro experiments on human cells are sometimes applicable to determining the expected outcomes of animal studies, there are often unexpected effects in animals, and whether these effects will be relevant to humans remains uncertain until clinical trials in human subjects have been performed. Thus, a more intensive human clinical trial may be required to testify the efficacy of renoprotective effects of DA extract in vivo.
